# Children’s Physiological and Perceptual Responses to Sports Exergames When Played in Different Positions

**DOI:** 10.3390/children10091489

**Published:** 2023-08-31

**Authors:** Nur Nashruha Mohd Sidek, Maziah Mat Rosly, Nasrul Anuar Abd Razak

**Affiliations:** 1 Department of Biomedical Engineering, Faculty of Engineering, Universiti Malaya, Kuala Lumpur 50603, Malaysia; 17177538@siswa.um.edu.my (N.N.M.S.); nasrul.anuar@um.edu.my (N.A.A.R.); 2 Department of Physiology, Faculty of Medicine, Universiti Malaya, Kuala Lumpur 50603, Malaysia

**Keywords:** active video game, adolescent, enjoyment, heart rate, perceived exertion

## Abstract

Today’s children are prone to becoming involved in exergames, but their positions during play have not been sufficiently investigated to determine whether the positions they adopt result in equal responses. The design of this study involved the collection of physiological and perceptual responses (i.e., heart rate (HR), rating of perceived exertion, and enjoyment score) during exergames in three different sports (bowling, tennis, and boxing) with players in different positions (sitting and standing). The participants played each game for 10 min while their HR was recorded. After the gameplay, each perceptual response was retrieved. The results revealed a significant increase in HR above rest during exergaming overall (*p* < 0.001). Standing gameplay resulted in a significantly higher HR (*p* < 0.001) than seated gameplay. Compared to tennis and bowling, boxing produced the highest physiological response (*p* < 0.001) and perceived exertion (*p* < 0.05) in both positions. The participants perceived all the sports exergames to be enjoyable, as their enjoyment scores did not significantly differ for each game (*p* > 0.5). For all the variables, no statistically significant differences between genders were identified (*p* > 0.5). This home-based intervention demonstrated that sports exergames are not only enjoyable; overall, they can provide at least moderately intense physical activity, whether played seated or standing.

## 1. Introduction

The evolution of exergaming technology has numerous benefits for physical health, resulting in significantly higher physical activity (PA) compared to sedentary behavior [1,2]. This is especially true for children and adolescents in home-based environments [3,4,5]. Exergames, which go beyond traditional hand-controller games, require children to move their entire bodies, providing a more active gaming experience [3]. They encompass various modalities, including aerobics, dance, balance, and other whole-body activities, aimed at boosting PA and enjoyment [6,7,8,9,10,11,12]. These games, particularly sports modalities, offer indoor alternatives for physical activity when outdoor activity is restricted, such as during the COVID-19 pandemic [5,13,14,15].

Active video games (AVGs) have the potential to increase PA levels, especially in children and teenagers, although the level of activity is comparable to traditional exercise [16]. Recent findings suggest that exergames generally provide light to moderate intensity and may not fulfill the recommended 60 min of moderate-vigorous PA per day [17]. Previously, seated video games were categorized as sedentary behavior due to the limited physical activity involved [18]. Consequently, video game play has become a global concern, as 43% of school-aged children reportedly spend over three hours per day on sedentary activities, including inactive video games [19]. The current understanding of specific intensity associations among various types of e-sports exergames based on the players’ corresponding positions remains limited, especially in children, calling for further investigation.

Exergame players tend to default to a standing position. A previous study indicated that standing exergames can provide high-intensity activity [20]. Both male and female participants showed significantly correlated heart rates (HR) and ratings of perceived exertion (RPE) while playing a boxing exergame in the standing position [21,22]. However, these findings were contradicted in a recent study [23], which found that seated exergames require more effort than standing exergames. Previous studies have also assessed the physiological cost of traditional video games in different positions (seated vs. standing) or during rest, comparing them directly to exergaming conditions that require a standing position [24,25,26,27]. However, the difference in energy expenditure between exergaming and traditional video games remains unknown, whether due to the physically interactive nature of exergaming or the fact that exergames are played while standing versus sitting [28]. This suggests that different positions during game-based exercises yield different physiological results.

Some randomized controlled trials have emphasized the use of accelerometers to determine PA levels during exergaming [29,30,31]. However, none of these studies monitored the intensity of PA during exergaming sessions, making the outcome measures somewhat unreliable in terms of exercise intensity, duration, and frequency. Additionally, the reliability of accelerometers for PA intensity classification in children compared to HR is questionable, given lower compliance rates and parental dependence [32,33,34,35]. In the context of e-sports exergaming, a significant correlation exists between HR measures and the intensity of PA; however, this correlation has not yet been comprehensively explored in relation to health and enjoyment.

Childhood is a crucial phase, as many modern non-communicable diseases are linked to sedentary lifestyles [36,37]. While most research on exergames has focused on adults [21,22,38] and the elderly [39,40], there is a lack of studies involving children and adolescents, particularly when comparing genders. Exergames are believed to be more enjoyable and preferable to traditional exercises, and this type of intervention can be easily performed even in limited spaces, such as at home. The quantification of PA levels during home-based exergaming can be enhanced by incorporating both objective measures, such as HR or perceptual exertion, and the perceived level of enjoyment. Moreover, it is anticipated that physiological and psychological exertions may vary between genders, thereby illuminating potential gender-specific preferences and outcomes within the context of home-based exergaming.

During periods of limited outdoor activity, such as during the pandemic, indoor exergames, especially those focused on sports, present an alternative avenue for maintaining PA. However, a clear understanding of how different types of sports exergames align with players’ movements during gameplay remains to be fully established. Additionally, an examination of the relationship between increased HR during these games and the corresponding level of physical effort is of interest. Another intriguing notion involves the integration of HR measurements with players’ self-reported perceptions, encompassing aspects like perceived exertion or enjoyment. This integrated approach holds the potential to offer valuable insights into the potential energy expended during exergaming within a home-based training program, conducted in sitting or standing positions. It also offers possible avenues for incorporating physical extra-curricular activities for school-going children that can be undertaken remotely, online, or through hybrid modes.

Therefore, this study aimed to: (1) investigate the physiological and perceptual responses to three different AVG sports games (bowling, tennis, and boxing) during gameplay in different positions (sitting and standing); and (2) compare the physiological and perceptual responses to home-based exergaming between the genders (male and female).

## 2. Materials and Methods

### 2.1. Participants

Fifty-one able-bodied children and adolescent males (*n* = 32; mean age = 11.3 SD 2.93 years) and females (*n* = 19; mean age = 11.2 SD 2.55 years) participated in this study (Table 1). A priori sample size was calculated (G*Power, version 3.1.9.7, Heinrich Heine University Düsseldorf, Düsseldorf, Germany) for an ANOVA analysis with an effect size of 0.25 and statistical power of 0.95. Due to the limited availability of related research, this statistical power study did not use data from previous studies. The sample size was set at 36 participants, but additional participants were recruited. The participants were recruited via acquaintances and word of mouth, and the intervention was undertaken in their own homes. All were eligible for inclusion if they met the following criteria: being aged between 7 and 17 years old; having no form of disability or disease; and being able to speak, write, and read either Malay or English. Participants with medical, cardiovascular, metabolic, and/or respiratory illnesses were not included in the study. Data collection was undertaken in a home-based setting. The study included all individuals able to provide written, informed parental consent. The study was approved on 11 September 2020 by the Human Research Ethics Committee at the University of Malaya (reference number: UMREC-995).

### 2.2. Methodology

**Figure 1:**
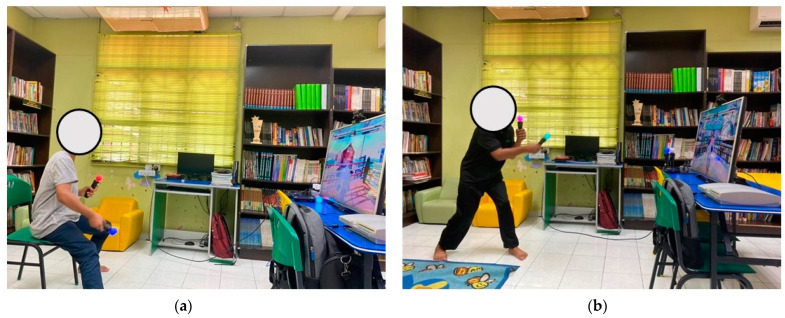
The player’s position during the sports exergaming intervention: the same participant is playing the sports AVG in two positions: (**a**) sitting and (**b**) standing.

The use of the Sony PlayStation Move enabled this sports exergames intervention. The equipment included the PlayStation 3 console, the Move eye camera, and the Move motion controller. This device is well-known for having the quickest response time while detecting movement interfaces [41]. The motion controller was calibrated to the eye camera depending on the player’s position in the gaming area. The players needed to be approximately 1.5 m away from the television screen to ensure a good interface during play (Figure 1). The Sports Champions 2 program was installed, and the games were then ready to be played.

**Figure 2:**
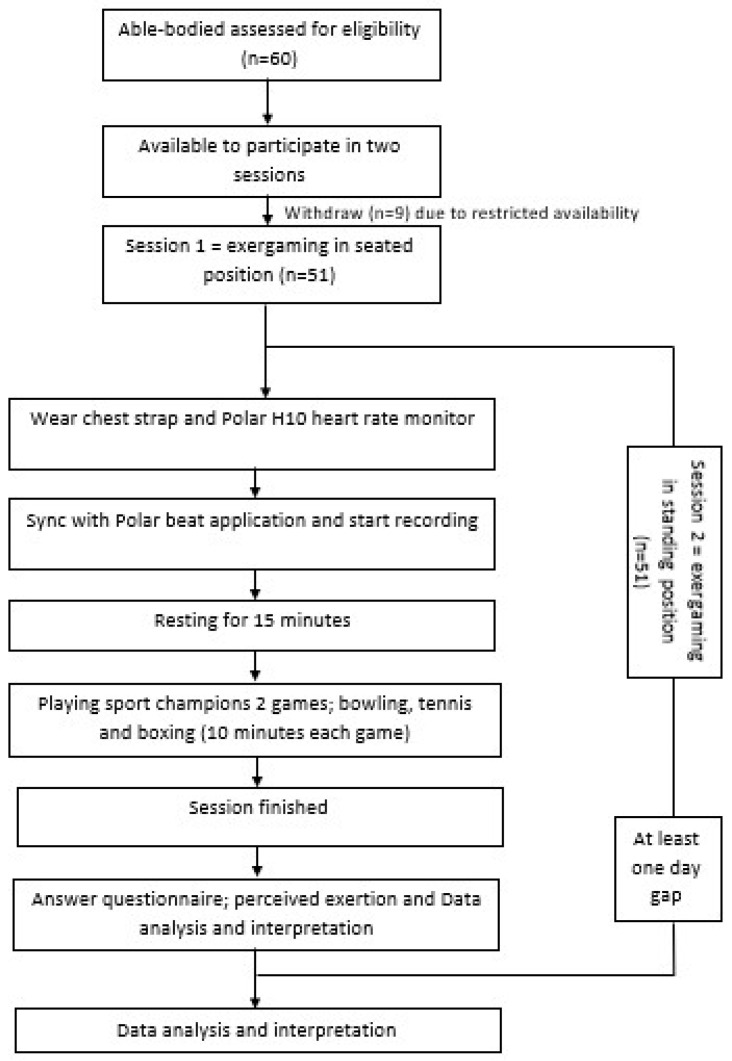
The flow diagram of the exergaming intervention.

The flow diagram of the protocol is shown in Figure 2. Before starting the exergames, the participants were provided with a 15 min familiarization session to practice the games and familiarize themselves with the Move motion controllers. A demonstration and instructions on playing the exergames were provided. To be consistent with other gaming studies [39,42], the participants completed the following four 10 min conditions: resting, Move bowling, Move tennis, and Move Boxing. The participants were each set up with a similar player avatar and level of difficulty (i.e., bronze mode). The game arrangement and player positions were not randomized to prevent energetic carryover between gaming configurations [43]. The participants needed to play rematches of the same game until they reached the end of the 10 min sessions. They were then allowed to rest for approximately 1–2 min. Their HR needed to decrease to 90 bpm or less before starting the next game [44]. After at least a one day gap, the same procedure was conducted with the players in a standing position.

#### 2.2.1. Heart Rate Measures

The HR was recorded continuously using a Polar H10^®^ Heart Sensor fixed to the participant’s chest and synced with the Polar Beat App (Polar Electro, Corp., Vantaa, Finland). The HR data were extracted and analyzed using Polar software (Polar FlowSync, version 3.0.0.1337), after which the data were imported into Microsoft Excel and SPSS for further analysis. This technology enabled the real-time tracking of each participant’s HR in five-second intervals. In a previous session, each student’s profile had been generated using the Polar Beat App, including information like their age, gender, height, weight, and predicted maximum HR. The predicted maximum HR was calculated using the Tanaka equation [45].

#### 2.2.2. Rating of Perceived Exertion

At the end of each game, the participants were asked to rate their subjective level of effort using the Borg original scale, RPE [46], and the Children’s OMNI Scale for walking/running (OMNI scale) [47]. The RPE described how much physical effort was exerted during a game (i.e., a rating of six indicates no effort at all, while a rating of 20 is associated with maximal effort). The RPE scale, which contains simplified wording, is a valid measure for quantifying the perception of physical exertion and the intensity of children’s exercise. It can also be applicable to a large number of participants over a short duration [48,49]. Meanwhile, the OMNI scale contains illustrated and verbal descriptions arranged across a numerical response range of 0 to 10 (i.e., a rating of zero refers to ‘not tired at all’ and a rating of nine is associated with ‘very, very tired’). The OMNI scale was validated against selected objective cardiorespiratory variables, revealing significant correlations with selected physiological variables for both male and female children [50].

#### 2.2.3. Enjoyment

The enjoyment of games was assessed using the Physical Activity Enjoyment Scale (PACES) [39]. The children were asked to rate their enjoyment based on a seven-point Likert scale (i.e., a rating of one meant they enjoyed the game, and a rating of seven meant they hated it). The total responses were then calculated, with the summation of each score ranging from five to 35. Research has demonstrated that the PACES has both reliability and validity in PA environments [51]. The scales were adapted to multiple languages (English and Malay) to accommodate the participants’ mother tongues.

### 2.3. Statistical Analysis

The results are presented as the mean ± standard deviation (SD). All the data were assessed for normal distribution using the Kolmogorov-Smirnov test. The RPE, OMNI, and PACES data were not normally distributed, so non-parametric statistical tests were performed. The HR values across the three sports exergames were compared using one-way analyses of variance (ANOVA) with Bonferroni post hoc comparisons. The RPE, OMNI, and PACES during different positions were analyzed using the Kruskal-Wallis H test. Paired *t*-tests were performed to determine any possible significant differences in HR during the gaming conditions. Meanwhile, the Wilcoxon Signed rank test was used to analyze differences in the RPE, OMNI, and PACES values obtained during the different games. The *p*-value was set at 0.05. The statistical analyses were conducted using SPSS version 26.0 (IBM SPSS Statistics for Windows, Version 26.0, Armonk, NY, USA: IBM Corp.). The effect size was calculated by dividing the difference of the means for the outcome variables by the pooled standard deviations. The interpretation was conducted in accordance with the Cohen guidelines, in which 0.20 is small, 0.50 is moderate, and 0.80 is large [52].

## 3. Results

The descriptive data regarding the height, weight, body mass index, and HR maximum predicted are presented in Table 1. The male group had a greater mean for all the variables compared to the female group. Thirty-six participants (70.6%) were considered underweight, ten (19.6%) were normal, four (7.8%) were overweight, and one (2%) was reported as obese, based on the body mass index-for-age definition; this is calculated as the weight in kilograms divided by the square of the height in meters and expressed relative to other children of the same sex and age.

The overall data for the physiological and perceptual responses are presented in Table 2. The Kolmogorov–Smirnov test results indicated that the HR values were normally distributed for all the games (*p* = 0.200). The perceptual response data, however, were not normally distributed (*p* < 0.05).

When the overall data were compared for all the participants in terms of their positions, significantly higher HR measurements were recorded during standing for all the games (*p* < 0.001; *p*_HR_ = 0.000028). Meanwhile, non-significant results were recorded for the perceptual response when comparing both positions, *p* > 0.05 (*p*_RPE_ = 0.575, *p*_OMNI_ = 0.728, *p*_PACES_ = 0.231). These statistics also indicated that sports exergames might provide moderate-intensity PA whether played while seated or standing, with %AVGHR > 55% of the HR maximum predicted and RPE > 13 [53].

### 3.1. Positional Differences

**Figure 3:**
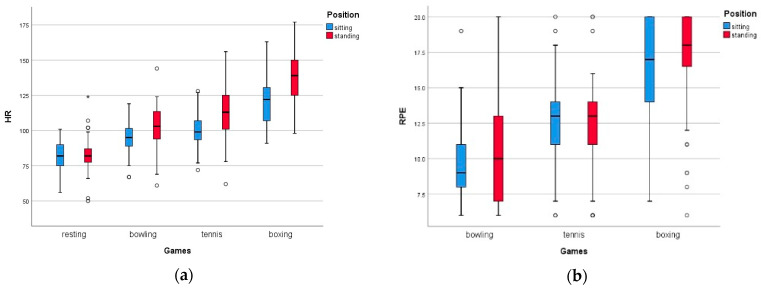
Comparison of variables during three different sports exergames. The mean values of (**a**) HR and (**b**) RPE against the sports exergames played in both positions, sitting, and standing.

In Table 3, the physiological and perceptual responses are presented according to the specific games and positions. All the HR data during the exergaming sessions differed significantly from the data obtained while resting (*p* < 0.01; *p*_bowling_ = 0.01, *p*_tennis_ = 1.27e^−4^, *p*_boxing_ = 2.65e^−10^), as shown in Figure 3. While seated, the HR values during boxing were significantly higher than when the tennis and bowling games were played (*p*_boxing-tennis_ = 1.50e^−16^, d_boxing-tennis_ = 0.27; *p*_boxing-bowling_ = 4.17e^−18^, d_boxing-bowling_ = 0.33). The HR values obtained during the tennis and bowling games also differed significantly (*p*_tennis-bowling_ = 0.20e^−5^, d_tennis-bowling_ = 0.43). Similarly, when participants were standing, the boxing game recorded significantly higher HR values compared to those recorded during the tennis and bowling games (*p*_boxing-tennis_ = 1.83e^−17^, d_boxing-tennis_ = 0.22; *p*_boxing-bowling_ = 8.08e^−22^, d_boxing-bowling_ = 0.35), while the rate during tennis was significantly higher than the rate during bowling (*p*_tennis-bowling_ = 2.11e^−12^, d = 0.34). The HR was measured as significantly higher while standing for all the games, *p* < 0.01 (*p*_bowling_ = 0.003, d_bowling_ = 0.10; *p*_tennis_ = 1.0e^−5^, d_tennis_ = 0.16; *p*_boxing_ = 3.80e^−5^, d_boxing_ = 0.13). The percentage of average HR from HRmax (%AVGHR) recorded that boxing while standing produced exertion of the highest value, 69.07% (SD 10.23), which corresponded to moderate-intensity exercise, according to Norton, Norton, and Sadgrove [53]. Meanwhile, the other games recorded a light-to-moderate intensity of PA, with a range of 47.11% (SD 5.81) to 56.05% (SD 8.21).

From the RPE data, the boxing games had significantly higher values than tennis or bowling, while tennis had significantly higher values than bowling, in both the sitting (*p*_boxing-tennis_ = 2.90e^−7^, d_boxing-tennis_ = 0.18; *p*_boxing-bowling_ = 3.05e^−9^, d_boxing-bowling_ = 0.53 [medium effect size]; *p*_tennis-bowling_ = 2.6e^−5^, d_tennis-bowling_ = 0.15) and standing positions (*p*_boxing-tennis_ = 1.83e^−17^, d = 0.22; *p*_boxing-tennis_ = 8.08e^−22^, d = 0.35; *p*_tennis-bowling_ = 2.11e^−12^, d_tennis-bowling_ = 0.34), as shown in Figure 3. The highest RPE score was recorded during the boxing game in the standing position, 17.08 (SD 3.69), which corresponded to ‘very hard’ exertion. Meanwhile, the other games, bowling (10.55 (SD 3.86)) and tennis (12.24 (SD 3.33)), corresponded to ‘fairly light’ and ‘somewhat hard’ exertion, respectively. When comparing the positions, no significant difference in the RPE values was identified in any of the games (*p*_bowling_ = 0.585, d_bowling_ = 0.03; *p*_tennis_ = 0.606, d_tennis_ = 0.02; *p*_boxing_ = 0.266, d_boxing_ = 0.03).

For the OMNI scale, the boxing game played in a seated position recorded a higher score than tennis and bowling, while tennis had a significantly higher value than bowling (*p*_boxing-tennis_ = 4.0e^−6^, d_boxing-tennis_ = 0.16; *p*_boxing-bowling_ = 2.24e^−8^, d_boxing-bowling_ = 0.26; *p*_tennis-bowling =_ 0.003, d_tennis-bowling_ = 0.09). In terms of the standing position, the OMNI scores were different for all the games (*p*_boxing-tennis_ = 3.18e^−9^, d_boxing-tennis_ = 0.26; *p*_boxing-bowling_ = 5.37e^−9^, d_boxing-bowling_ = 0.30; *p*_tennis-bowling_ = 0.028, d_tennis-bowling_ = 0.05). When comparing the positions, no difference was identified in the OMNI scores for any of the games (*p*_bowling_ = 0.919, d_bowling_ = 0.003; *p*_tennis_ = 0.340, d_tennis_ = 0.03; *p*_boxing_ = 0.065, d_boxing_ = 0.05). The highest score was during the boxing game while standing, 7.98 (SD 2.58), which corresponded approximately to ‘really tired.’

For the PACES scale, no significant differences were found between any of the games in either position (*p*_bowling_ = 0.255, d_bowling_ = 0.03; *p*_tennis_ = 0.372, d_tennis_ = 0.02; *p*_boxing_ = 0.835). It can be assumed that all the games produced the same level of enjoyment, for which the percentage score ratings ranged from the lowest of 77.37 (SD 19.14) to the highest of 83.31 (SD 19.29), irrespective of the position. However, in the seated position, the PACES scores for the tennis and boxing games differed significantly (*p*_boxing-tennis_ = 0.011, d_boxing-tennis_ = 0.04); however, these discrepancies were disregarded due to the small effect size.

### 3.2. Gender Gap

This study demonstrated no significant difference between the genders with respect to the HR, RPE, OMNI, and PACES scores. Based on the results, the genders did not differ significantly in terms of HR, as shown in Table 4. However, higher HR values were recorded among the female participants for all the games in both positions, except for boxing in the standing position. The same applied to the %AVGHR, whereby the male participants recorded higher percentages than the females in the boxing game while standing.

**Figure 4:**
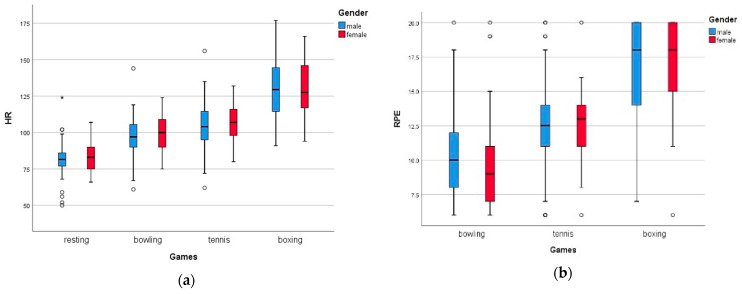
Graph showing (**a**) HR and (**b**) RPE against the game played for male and female participants.

Moreover, for both male and female participants, the RPE and OMNI values were significantly higher for boxing than for tennis and bowling. The RPE for tennis was higher than that for the bowling game in all cases, except for the male participants while standing. Male participants recorded that tennis required greater OMNI exertion than bowling in the seated position, while female participants perceived this while standing to play the games. Nevertheless, males found that boxing in a sitting position was more enjoyable compared to the other conditions, approximately 85%, based on the PACES scores. Regardless, no statistically significant differences were identified between the genders for any variables (*p* > 0.5), as shown in Figure 4.

## 4. Discussion

The main findings of this study revealed a significant increase in HR above rest during the three games: bowling, tennis, and boxing. There was a significant HR increase when the exergames were conducted in a standing position. The boxing game recorded the highest HR reading in both positions compared to the other games, bowling and tennis. However, when comparing the gender groups, no significant interaction was identified between those games played in different postures. The perceptual responses recorded during the boxing games were higher than those recorded during tennis and bowling, and the responses differed significantly. When comparing the positions, it was observed that the participants scored higher for RPE, OMNI, and PACES during standing gameplay. However, no significant interaction was identified between those perceptual responses and the games or the position adopted while exergaming.

The purpose of this study was to compare the three sports exergames. Overall, the modalities of the three exergames varied, ranging from moderate to vigorous intensity [22,44,54]. Boxing was the only game of the three that demanded bilateral upper extremity movement and produced greater movement quantity [55]. Boxing is thought to cause the greatest energy expenditure in adolescents due to the highest center of pressure movement [56]. This explains why, when compared to bowling and tennis, boxing caused the greatest physiological reaction. Meanwhile, the tennis game required more specific tennis motor skills (such as cognitive control for efficient decision-making together with visual processing) [57], which resulted in more detailed movement during gameplay. As can be observed, the participants took longer to grasp the game and adopt an effective technique for returning their opponents’ serves. Because the bowling game was only played individually, there was no avatar opponent, so the participants discovered that scoring and winning for each trial were easier, so they played more leniently. This clarifies the substantial differences between the three sports exergames.

Boys are often recognized as being more physically active than girls [58,59], as supported by a previous study that found higher energy consumption in boys during AVG sessions, specifically in tennis games [56]. However, the results of this study were not influenced by gender. This is consistent with the recent study that indicates that gender did not have an influence on the total physical activity (PA) time during the 30 min Wii games (Wii Fit, Just Dance, and Wii Sports) sessions in a school-based intervention that lasted for a total of 18 weeks [60]. Furthermore, no significant differences in PA enjoyment scales were found between genders during Nintendo Wii AVG sessions in a laboratory setting for a 12-week intervention [10]. Similarly, the differences in perceived enjoyment over time did not show a significant effect on gender during ExerCube sessions [61]. These findings suggest that exergaming developers aim to create games that appeal to both genders, which may explain the lack of gender differences in PA engagement and enjoyment.

These observations are promising as they indicate that health professionals may be able to use exergaming as an appropriate PA promotion strategy for all children, regardless of their gender [60]. It is worth noting that in this study, female participants achieved higher physiological responses, possibly due to their perceived comfort in engaging in sports exergames in the home environment compared to a public space. Similarly, previous studies have found that girls are more likely to be active in exergaming sessions [62], a finding that holds true even in a home-based environment [63]. Overall, these findings support the use of exergaming as an inclusive PA promotion strategy for children, irrespective of their gender.

Enjoyment is indeed associated with several physical activity (PA) benefits. The PACES questionnaire has proven to be a valuable tool for assessing PA enjoyment due to its structural validity and internal consistency, particularly among young children [51]. In this study, the participants reported equal enjoyment of all the games, with PACES scores exceeding 77%. They expressed enjoyment, liking, and having a lot of fun during gameplay, without feeling annoyed by the games. This was especially true for first-time users of exergaming, such as children who had never owned an active video game (AVG) before. However, as children become more engaged in exergaming, their perceptions of a game’s intensity may change, along with developing greater enthusiasm for play and exercise over time [39,44].

Nevertheless, the most recent study on exergames intervention using the Exercube [60] demonstrated no significant differences in perceived enjoyment (*p* = 0.164) after two and 12 weeks of intervention. The mean score on the PACES changed from 71.3 ± 6.3 in week two to 62.4 ± 14.2 in week 12, indicating a decrease in perceived enjoyment over time. However, the effect size was small (f = 0.073) [61]. These findings are relevant, as a decline in physical activity can be observed, especially during prepubertal age and early puberty, which is often associated with a lack of perceived enjoyment in PA.

While most of the previous interventions were conducted in laboratory-based environments, this study implemented considerably more natural experiences for participants playing exergames at home. Additionally, the sports exergames were found to be fun and enjoyable for the children. These measures provide information to parents, especially in recommending their children engage in sports exergames of adequate duration daily in order to stay physically active [64], whether they are playing seated or in a standing position.

It is important to note the limitations of the study. The order of the games was not randomized for each session due to the limited availability of the children’s schedules as the gaming was conducted at home. Next, the chest strap used in this study was not customized in size for children or adolescents. As only the smallest size of adult chest strap was available, it was adjusted to fit the generally smaller body sizes of the participants (70.6% of the participants were underweight). Lastly, the individual’s previous experience of exergaming was not taken into consideration. This may or may not have influenced the variables, as a skilled player may have performed better in a game compared to a first-time player.

As this study only investigated the physiological responses in different positions (sitting and standing), no deeper biomechanical statistics were proven, as such activities can only be performed in a laboratory setting. It is recommended to conduct further analysis on the biomechanics of positional differences during exergaming as this would provide feedback on the influence of gameplay and energy expenditure at the body segment level (i.e., Kinect sensor) [65]. This would support the findings, which indicate exergaming is better played while standing, not only through objectively measured analysis. Exergaming would also be useful to integrate into physical education or extracurricular activities among predominantly sedentary children, especially in developing countries [66]. Finally, rather than focusing on obesity cases as many previous studies have undertaken, future work might be targeted at an underserved demographic (the underweight) to investigate whether exergames can lead to increased PA.

## 5. Conclusions

This study demonstrated that sports exergames can provide multi-level PA intensities with at least moderate intensity identified in the players of the three sports games used. Higher levels of intensity and physiological response can be achieved when exergames are played in a standing position. The perceptual responses to these games did not differ in either position. These measures may provide information to parents, especially in terms of recommending their children engage in sports exergames of an adequate duration daily in order to stay physically active, whether they play in a seated or standing position. This study also found no gender bias when sports exergames were conducted in a home-based environment.

## Figures and Tables

**Table 1:** Demographic data of the participants.

Characteristics	All (*n* = 51)		Male (*n* = 32)		Female (*n* = 19)	
	Mean	SD	Mean	SD	Mean	SD
Age (years)	11.24	2.77	11.25	2.93	11.21	2.55
Weight (kg)	37.23	17.17	39.85	20.46	32.82	8.06
Height (m)	1.41	0.16	1.41	0.19	1.39	0.12
BMI (kg/m^2^)	17.99	4.73	18.80	5.61	16.61	2.23
HRmax predicted (bpm)	200.12	1.90	200.09	2.02	200.16	1.74

BMI: body mass index; HR: heart rate; bpm: beats per minute.

**Table 2:** The overall data of all participants playing the sports exergames in sitting and standing positions.

Variables	Sitting	Standing	All	F	*p*
HR (bpm)	104.99 ± 18.40	117.62 ± 23.15 *	111.31 ± 21.81	27.90	<0.000
%AVGHR	52.45 9.14	58.75 11.44 *	55.60 10.81	28.30	<0.000
RPE	12.97 ± 4.11	13.29 ± 4.56	13.13 ± 4.33	0.42	0.519
OMNI	4.65 ± 3.17	4.82 ± 3.33	4.74 ± 3.25	0.91	0.660
PACES	28.08 ± 6.15	28.79 ± 6.21	28.43 ± 6.18	1.02	0.314

HR: heart rate; bpm: beats per minute; %AVGHR: percentage average heart rate; RPE: rating of perceived exertion; OMNI: children’s OMNI perceived scale; PACES: physical activity enjoyment scale; * *p* < 0.001: significant difference from sitting position.

**Table 3:** The data for variables for each sport, exergame, and position.

Variables	Position	Rest	Bowling	Tennis	Boxing
HR (bpm)	Seated	81.94 ± 9.32	94.29 ± 11.78	98.88 ± 11.83 ^a^	121.80 ± 17.52 ^a,b^
	Standing	82.43 ±12.11	102.35 ± 14.42 ^c^	112.22 ± 16.71 ^ac^	138.29 ± 20.94 ^a,b,c^
%AVGHR	Seated	40.94 ± 4.60	47.11 ± 5.81	49.39 ± 5.79 ^a^	60.85 ± 8.71 ^a,b^
	Standing	41.18 ± 5.99	51.13 ± 7.14 ^c^	56.05 ± 8.21 ^a^	69.07 ± 10.23 ^a,b^
RPE	Seated	-	9.88 ± 2.85	12.65 ± 2.99 ^a^	16.37 ± 3.55 ^a,b^
	Standing	-	10.55 ± 3.86	12.24 ± 3.33 ^a^	17.08 ± 3.69 ^a,b^
OMNI	Seated	-	2.73 ± 2.38	4.18 ± 2.57 ^a^	7.06 ± 2.87 ^a,b^
	Standing	-	2.78 ± 2.40	3.69 ± 2.36	7.98 ± 2.58 ^a,b^
%PACES	Seated	-	79.99 ± 14.99	77.37 ± 19.14	83.31 ± 18.12
	Standing	-	82.97 ± 15.23	80.50 ± 18.68	83.31 ± 19.29

HR: heart rate; bpm: beats per minute; %AVGHR: percentage average heart rate; RPE: rating of perceived exertion; OMNI: children’s OMNI perceived scale; PACES: physical activity enjoyment scale; ^a^ *p* < 0.001: significantly higher from bowling; ^b^ *p* < 0.001: significantly higher from tennis; ^c^ *p* < 0.01: significantly different from sitting position.

**Table 4:** The data between genders playing the three sports exergames in sitting and standing positions.

Variables	Position	Sexes	Rest	Bowling	Tennis	Boxing
HR	Seated	Male	80.91 ± 9.54	93.44 ± 12.04 ^a^	97.25 ± 11.86 ^a^	120.94 ± 17.56 ^a,b,d^
		Female	83.68 ± 8.90	95.74 ± 11.52 ^a^	101.63 ± 11.57 ^a^	123.26 ± 17.84 ^a,b,d^
	Standing	Male	81.94 ± 9.32	101.69 ± 15.22 ^a^	112.06 ± 18.38 ^a,b^	139.41 ± 21.54 ^a,b,d^
		Female	83.47 ± 10.50	103.47 ± 13.28 ^a^	112.47 13.92 ^a^	136.42 ± 20.33 ^a,b,d^
%AVGHR	Seated	Male	40.42 ± 4.68	46.67 ± 5.86 ^a^	48.56 ± 5.64 ^a^	60.40 ± 8.55 ^a,b,d^
		Female	41.81 ± 4.44	47.84 ± 5.81 ^a^	50.79 ± 5.92 ^a^	61.62 ± 9.16 ^a,b,d^
	Standing	Male	40.86 ± 6.43	50.79 ± 7.47 ^a^	55.96 ± 8.97 ^a,b^	69.61 ± 10.41 ^a,b,d^
		Female	41.71 ± 5.30	51.71 ± 6.70 ^a^	56.20 ± 6.99 ^a^	68.16 ± 10.12 ^a,b,d^
RPE	Seated	Male	-	10.16 ± 2.60	12.97 ± 3.31 ^c^	16.25 ± 3.82 ^c,e^
		Female	-	9.42 ± 3.25	12.11 ± 2.33 ^c^	16.58 ± 3.13 ^c,e^
	Standing	Male	-	10.63 ± 3.79	11.75 ± 3.65	17.00 ± 3.66 ^c,e^
		Female	-	10.42 ± 4.09	13.05 ± 2.59 ^c^	17.21 ± 3.84 ^c,e^
OMNI	Seated	Male	-	2.88 ± 2.47	4.38 ± 2.39 ^c^	6.69 ± 3.17 ^c,e^
		Female	-	2.47 ± 2.27	3.84 ± 2.87	7.68 ± 2.24 ^c,e^
	Standing	Male	-	3.06 ± 2.24	3.22 ± 2.18	7.75 ± 2.65 ^c,e^
		Female	-	2.32 ± 2.65	4.47 ± 2.50 ^c^	8.37 ± 2.48 ^c,e^
PACES	Seated	Male	-	28.50 ± 5.01	26.44 ± 7.66	29.72 ± 5.38 ^e^
		Female	-	27.16 ± 5.66	28.16 ± 4.65	28.21 ± 7.77
	Standing	Male	-	29.38 ± 5.37	27.53 ± 7.35	29.5 ± 6.74
		Female	-	28.47 ± 5.36	29.26 ± 4.87	28.58 ± 6.93
%PACES	Seated	Male	-	81.43 ± 14.31	75.54 ± 21.89	84.91 ± 15.37 ^e^
		Female	-	77.59 ± 16.17	80.45 ± 13.27	80.60 ± 22.21
	Standing	Male	-	83.93 ± 15.34	78.66 ± 20.99	84.29 ± 19.24
		Female	-	81.35 ± 15.31	83.61 ± 13.93	81.65 ± 19.80

HR: heart rate; bpm: beats per minute; %AVGHR: percentage average heart rate; RPE: rating of perceived exertion; OMNI: children’s OMNI perceived scale; PACES: physical activity enjoyment scale; ^a^ *p* < 0.001: significantly different from resting; ^b^ *p* < 0.001 and ^c^ *p* < 0.05: significantly higher from bowling; ^d^ *p* < 0.001 and ^e^ *p* < 0.05: significantly higher from tennis.

## Data Availability

Data is not available due to privacy and ethical restrictions.
